# *BbCFEM7* plays an important role in the pathogenicity and gut microbial community formation in the co-infection of *Beauveria bassiana* with *Metarhizium rileyi*

**DOI:** 10.1128/spectrum.02457-25

**Published:** 2026-01-06

**Authors:** Xu Zhang, Tianjiao Zhan, Qingqing Liu, Mengfei Xing, Wangjiao Yu, Bin Chen, Yuejin Peng

**Affiliations:** 1 College of Plant Protection in Yunnan Agricultural University, Kunming, Yunnan, China; China Agricultural University, Beijing, China

**Keywords:** CFEM domain, *Beauveria bassiana*, *Metarhizium rileyi*, gut microbe, Enterococcus, pathogenic fungi-gut bacteria interaction

## Abstract

This study investigated the effects of the *BbCFEM7* gene in *Beauveria bassiana* on host pathogenicity and the gut microbial community during co-infection with Metarhizium rileyi in *Spodoptera litura*. Our results showed that *BbCFEM7* significantly enhanced the virulence of *B. bassiana* and significantly reduced the host LT_50_ in the 1:0, 9:1, and 1:1 (Bb:Mr) groups. Sequencing of the gut microbial diversity showed that the presence of BbCFEM7 significantly altered the structure of the host gut microbial community, particularly in the 9:1 group. The presence of *BbCFEM7* increased the relative abundance of Enterococcus and decreased that of Thomasclavelia. Furthermore, *BbCFEM7* was found to play a pivotal role in enhancing *B. bassiana*’s tolerance to chemical stress and promoting its rapid colonization of the host’s hemolymph, thereby giving it an advantage over *M. rileyi*. These results suggest that *BbCFEM7* plays an important role not only in fungal pathogenicity but also in indirectly influencing host health by regulating gut microbial communities. This study provides a new perspective on the interactions between entomopathogenic fungi, hosts, and gut microorganisms and establishes a theoretical basis for optimizing the application of entomopathogenic fungi in pest control.

**IMPORTANCE:**

This study investigates the role of *BbCFEM7* in *B. bassiana* during co-infection with *M. rileyi* in *S. litura*. It reveals that *BbCFEM7* significantly enhances fungal pathogenicity, shortens the time taken for the host to die, and alters gut microbial communities. These findings provide novel insights into the interactions between entomopathogenic fungi, hosts, and gut microbes, offering a theoretical basis for optimizing the application of entomopathogenic fungi in pest control, while also contributing to our understanding of the insect microbiome and immunology.

## INTRODUCTION

Gut microbes are recognized as one of the protective barriers of arthropods, which play a key role in the physiology, ecology, and evolution of insects, as well as in nutrient metabolism, immune defense, behavioral regulation, adaptive evolution, competition with other pathogenic microorganisms, and niche differentiation of insects (1, 2). However, the competitive relationship between gut microbes and pathogens is complex. Due to the diversity of arthropods and pathogens, research in this field remains a serious challenge. Although Zaneveld et al. have summarized the changes of the host gut microbial community with the Anna Karenina principle (3), there are still many questions about the underlying mechanism. Some gut microbes can inhibit the invasion of pathogens (symbiotic microorganisms) (4, 5) or accelerate the death of the host (opportunistic pathogens) by occupying the gut niche, consuming nutrients, or secreting antimicrobial substances (6).

*B. bassiana* is a widely studied entomopathogenic fungus that has to go through an infection cycle before it infects and kills its host. The conidia of fungi attach to the surface of the insect body and form a bud tube through germination under suitable temperature and humidity conditions (7, 8). After passing through the body wall and entering the insect, the bud tube rapidly grows into the mycelium and spreads in the hemolymph of the insect. The mycelium absorbs nutrients from the insect, such as water, proteins, and sugars, while producing toxins (such as beauverine) (9) that cause physiological disorders in the insect. As the mycelium multiplies, the tissues inside the insect are destroyed and die. When the insect dies, the mycelium penetrates the surface of the insect again, forming an airborne mycelium and conidial stalk, releasing new conidia, which will enter the next cycle of infection under the right conditions (8).

In the process of proliferation in the host, the competition mechanism between the fungus and the host is complex and diverse. Insect-pathogenic fungi do not exist in isolation within natural environments. Their infection processes are profoundly influenced by the symbiotic microbial communities residing on and within the insect’s body surface. For example, when *M. rileyi* infects cotton bollworm, the host secretes antimicrobial peptides (AMPs) to counter the proliferation of pathogenic fungi in the hemolymph (10). The gut microbiome, as one of the defensive barriers of insects, also takes some measures to defend itself. For example, the composition and function of gut microbes can change dramatically, and some are able to defend against infection by activating the host immune response or directly inhibiting the growth of pathogenic bacteria. The presence of *Acinetobacter baumannii* makes the insects more resistant to *Metarhizium anisopliae* (11).

Common in fungal extracellular membrane (CFEM) domain proteins are unique to fungi, which play an important role in the process of obtaining iron for fungi. In entomopathogenetic fungi, Peng et al. systematically revealed the role of CFEM protein in iron acquisition in *B. bassiana* (12). These proteins are not only involved in obtaining iron from heme and trivalent iron ions but are also essential for the entire life cycle of *B. bassiana*, including saprophytic and pathogenic growth. In addition, they reported for the first time the synergistic division of labor and compensation of CFEM family genes at different levels of iron starvation. In *Metarhizium robertsii*, the CFEM domain protein (Mcdc9) acts as a contact elicitor to induce hygienic behavior in flies against fungal parasite infection (13). In *Metarhizium acridum*, the absence of *MaCFEM1* leads to rapid proliferation of opportunistic bacteria in the insect host’s gut, resulting in rapid insect death (14). *BbCFEM7* proved to be an important virulence factor. However, what role *BbCFEM7* plays in host hemolymph proliferation is unknown.

In this study, different proportions of *B. bassiana* and *M. rileyi* were combined and verified by gut microbial diversity sequencing and related *in vivo* and *in vitro* experiments to investigate the role of two entomopathogenic fungi in infecting *S. litura*. The effects of CFEM domain proteins on fungal virulence and gut microbial community structure reveal the functions of CFEM proteins in fungal host and gut microbial interactions, providing a new perspective for understanding the ecological relationship between insect health and pathogenic bacterial infection. In addition, the results help optimize the use of entomopathogenic fungi, improve their application efficiency in pest control, and provide new research directions for insect microbiome and immunology research.

## MATERIALS AND METHODS

### Strains and media

*M. rileyi* strain XSBN200920 and *B. bassiana* 2860 (wild-type [WT] and Δ*BbCFEM7* mutants) were utilized (12, 15) as previously described. For culturing, Sabouraud Maltose Agar medium plates (SMAY: 1% peptone, 1% yeast extract, 4% maltose, and 1.5% agarose) and Sabouraud Dextrose Agar medium plates (SDAY: 1% peptone, 1% yeast extract, 4% glucose, and 1.5% agar) were used. These fungi were incubated at 25°C under a 12-h light/12-h dark cycle in a culture chamber (RG-300, Xi’an Hengli Instrumentation Co., Ltd.). Conidial suspensions were prepared from fungi grown on SMAY and SDAY plates for 10 days.

### Insect feeding

*S. litura* is raised indoors in an artificial climate room with a temperature set at 25°C, a photoperiod of 16 h light: 8 h darkness, and a relative humidity of 70% (16). The larvae are provided with fresh artificial feed every day. For adult rearing, the adults are placed in a 50 × 50 × 50 cm cage and provided with fresh honey water every other day.

### Bioassay

To determine the virulence, fifth-instar *S. litura* larvae cultured over five generations were used for blood cavity injection experiments. A 1 × 10⁵ conidia/mL fungal conidial suspension was used to infect *S. litura* larvae, including *M. rileyi* and *B. bassiana*, according to the previous method. *M. rileyi* XSBN200920 was combined with WT or Δ*BbCFEM7* mutants and set to five ratios (1:0, 9:1, 1:1, 1:9, and 0:1 [V:V]). Five microliters of spore suspension was injected into the blood cavity of each larva in each group. The experiment was repeated three times, with no fewer than 35 samples treated each time. After treatment, the larvae were raised indoors in an artificial climate. Mortality was monitored daily, and dead larvae were placed in Petri dishes to observe humidity and confirm whether they were infected by the tested strain. The dead insects were placed in Petri dishes for about 10 days and photographed (15).

### Physiology experiments

A conidial suspension consisting of 2.5 μL 1 × 10^7^ conidia/mL was obtained from *M. rileyi* strain XSBN200920 and *B. bassiana* strains (WT and Δ*BbCFEM7* mutants), respectively. The inoculum was added with the Czapek's agar (CZA) medium containing 50 μg/mL Congo red, 0.03 mM/L menadione, and 3 mM/L H_₂_O_₂_. The plate was cultured at 25°C, with 12 h of light/12 h of darkness for 7 days, and the colony diameter was measured. In the plate antagonism experiment of *M. rileyi* and *B. bassiana*, the SMAY plate was used as the vegetative growth medium for both strains. A fungal conidial suspension of 2.5 μL 1 × 10^7^ conidia/mL was inoculated on a plate, and photographs were taken on day 7. Conidia of the two strains were inoculated at a distance of 1.5 cm. The experiment was repeated three times with three parallel controls each time.

### Morphological observation of fungi in hemolymph

To observe mycelium growth in insects, 5 microliters of 1 × 10⁵ conidia/mL conidial suspension was injected into the blood cavity of the host. The larvae were killed some time after injection (hours post-infection [HPI]), and their hemolymph was diluted 1:1 in a sterile anticoagulant (0.14 M NaCl, 0.1 M glucose, 25 mM sodium citrate, and 30 mM citric acid). The mycelium is then photographed under a microscope and viewed (17).

### Gut microbial diversity sequencing

Referring to the previous method (15), a suspension of 1 × 10⁵ conidia/mL was injected into *S. litura* 5th-instar larvae. The injection procedures and specific operations are the same as those described in Section 2.3. On day 5 after injection, the intestines of the injected larvae were carefully dissected, and samples were sent to Majorbio Co. Ltd. (Shanghai) for paired terminal sequencing on the Illumina MiSeq PE300 platform (Illumina, San Diego, USA). Each fungus-treated sample was set up with three replicates, with each replicate containing six larvae. The Majorbio Cloud platform (https://cloud.majorbio.com) is used for gut microbiota bioinformatics analysis. Based on operational taxonomic units (OTUs), the dilution curve and α diversity index were generated, including the observed OTUs. Kruskal-Wallis rank sum test was used to compare the relative abundance of gut microbes at phylum and genus levels among the groups. α diversity-Chao richness estimates reflecting species richness and community diversity were calculated using QIIME2 (https://qiime2.org/). Principal component analysis (PCA) based on Bray-Curtis differences was used to assess the similarity of microbial communities in different samples using Vegan v2.5-3 packages. Linear discriminant analysis (LDA ≥ 2, *P* < 0.05) was used to identify species with significant differences in sample classification.

### Data analysis

Data conforming to normal distribution and homogeneity of variance between two groups were compared using an unpaired *t*-test. Comparisons among multiple groups were conducted via a univariate analysis of variance (ANOVA) and bivariate ANOVA, followed by Tukey’s honest significance difference test (Tukey’s HSD). A *P*-value of < 0.05 was considered statistically significant. The software used is GraphPad Prism 7.00.

## RESULTS

### Virulence of *BbCFEM7* in host co-infection of *B. bassiana* and *M. rileyi*

**Fig 1:**
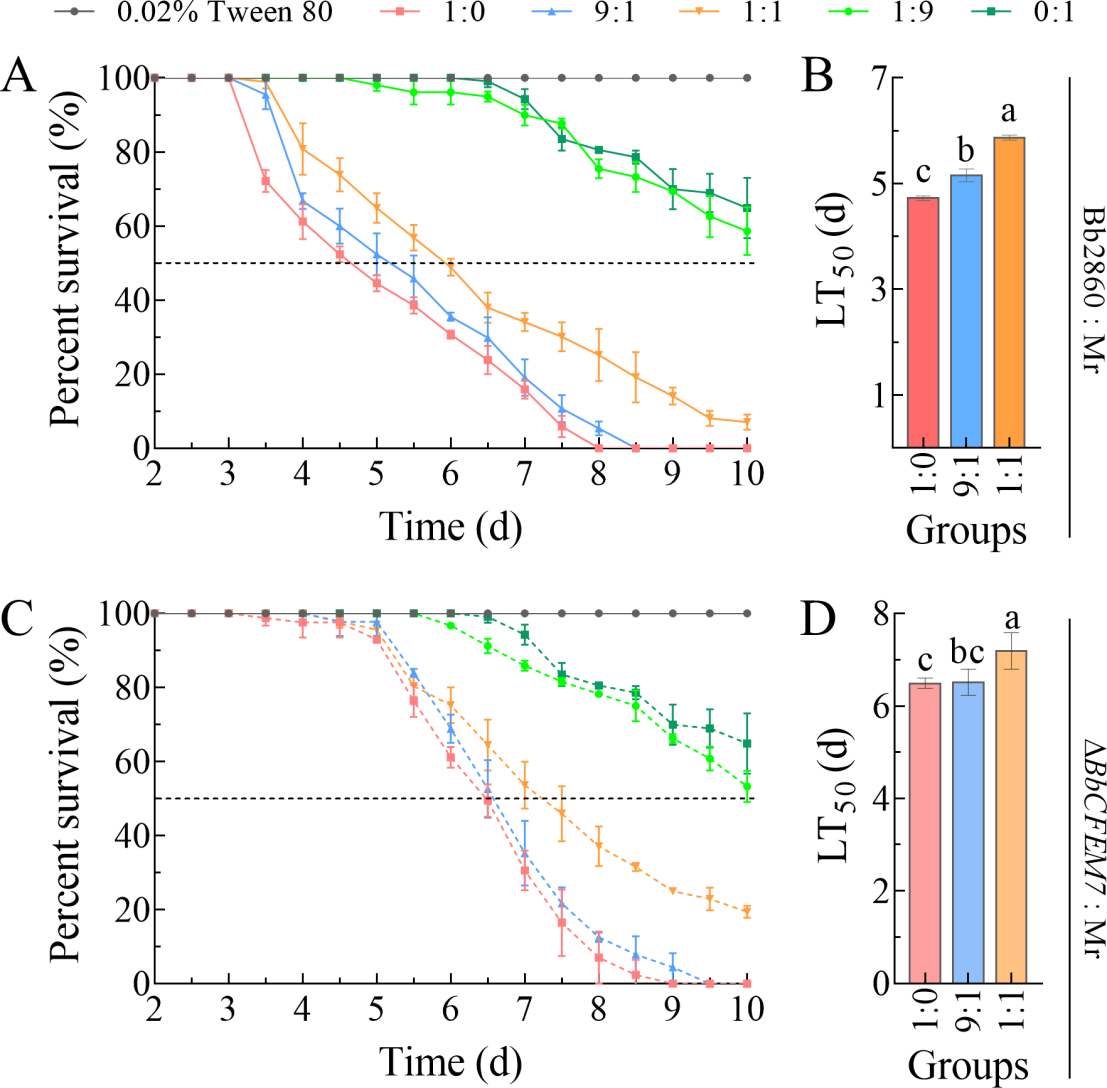
Virulence of *BbCFEM7* in host co-infection of *B. bassiana* and *M. rileyi*. (**A and B**): Larva survival curves and LT_50_ of *B. bassiana* 2860 (WT) and *M. rileyi* XSBN200920 conidia suspensions at 1 × 10^5^ conidia /mL under five groups of infection, respectively. Larva survival curves and LT_50_ of (**C and D**): the Δ*BbCFEM7* mutant and *M. rileyi* XSBN200920 conidia suspension under 1 × 10^5^ conidia /mL infection in five groups, respectively. Categories include Bb:Mr (1:0, 9:1, 1:1, 1:9, and 0:1). Tukey’s HSD test: *P* < 0.05. Error bars represent standard deviation.

The virulence of *B. bassiana* (WT and ΔBbCFEM7 mutants) and *M. rileyi*, alone or in combination, was assessed by injection into larvae. Survival curves and half-lethal times (LT_50_) were analyzed (Fig. 1).The results showed that the presence of *BbCFEM7* significantly affected the virulence of *B. bassiana* co-infected with *M. rileyi*. Specifically, compared with the Δ*BbCFEM7* mutant, the survival curve of larvae infected with *B. bassiana* WT decreased more sharply (Fig. 1A and C), and the LT_50_ was significantly smaller (Fig. 1B and D). This observation was further supported by LT_50_ values, with WT strains dying faster than mutant strains in all test ratios (1:0, 9:1, and 1:1) (*P* = 0.0000008, *P* = 0.0000132, and *P* = 0.0000173). By comparing the larval survival rate and LT_50_ under different combination ratios, the role of the *BbCFEM7* gene in the co-infection of *B. bassiana* and *M. rileyi* and its influence on the pathogenicity of the host can be evaluated.

### α and β analyses of host gut microbial diversity by *BbCFEM7* during co-infection

**Fig 2:**
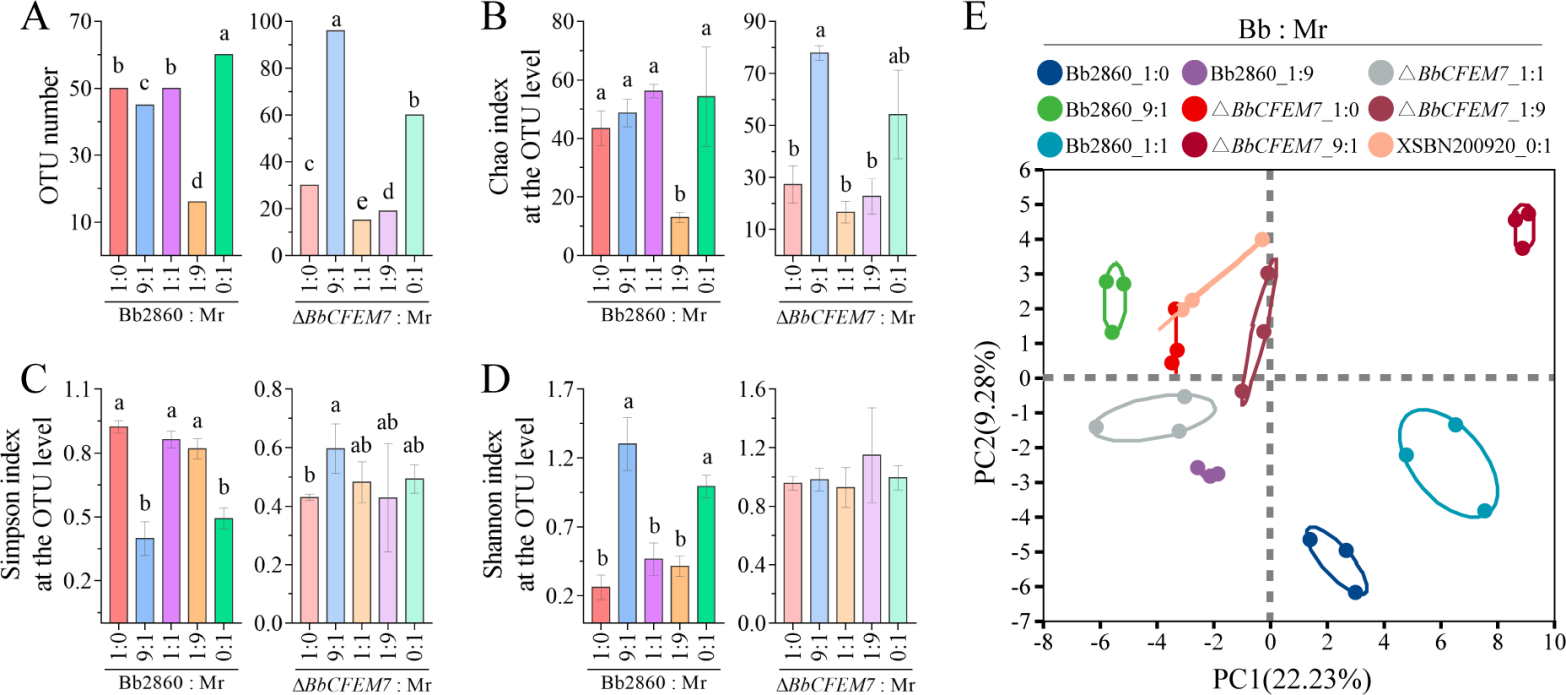
α and β analyses of host gut microbial diversity by *BbCFEM7* during co-infection with *B. bassiana* and *M. rileyi*. (**A–D**): OTU index (**A**), Chao Index (**B**), Simpson Index (**C**), and Shannon Index (**D**). a, b, and c denote statistically significant differences between groups. (**E**) PCoA of the gut microbial β diversity index following co-infection with WT and Δ*BbCFEM7* mutants of *B. bassiana*, after treatment with a 1 × 10^5^ conidia/mL suspension for 3 days. Statistical significance tested using Tukey’s HSD test: *P* < 0.05. Error bars represent standard deviation.

The diversity of gut microbiota was assessed during co-infection with *B. bassiana* (both WT and Δ*BbCFEM7* mutant) and *M. rileyi* (Fig. 2). Changes in the gut microbial communities were quantified using α-diversity indices, which include OTU), species richness (Chao index), evenness (Simpson index), and diversity (Shannon index) (Fig. 2A through D). The results indicated significant differences between the WT and Δ*BbCFEM7* mutant groups, demonstrating that *BbCFEM7* influences both the abundance and evenness of the gut microbiota. For instance, larvae infected with the WT strain exhibited a higher Chao index compared to those infected with the Δ*BbCFEM7* mutant, suggesting enhanced species richness due to the presence of *BbCFEM7* (Fig. 2B). Additionally, a β-diversity analysis, employing principal coordinate analysis (PCoA) based on Bray-Curtis dissimilarity, showed distinct clustering patterns of the gut microbiome between the WT and mutant groups (Fig. 2E). This analysis confirms that *BbCFEM7* significantly modifies the overall structure of the gut microbiome during co-infection.

### Effects of *BbCFEM7* on gut microbial composition during co-infection

**Fig 3:**
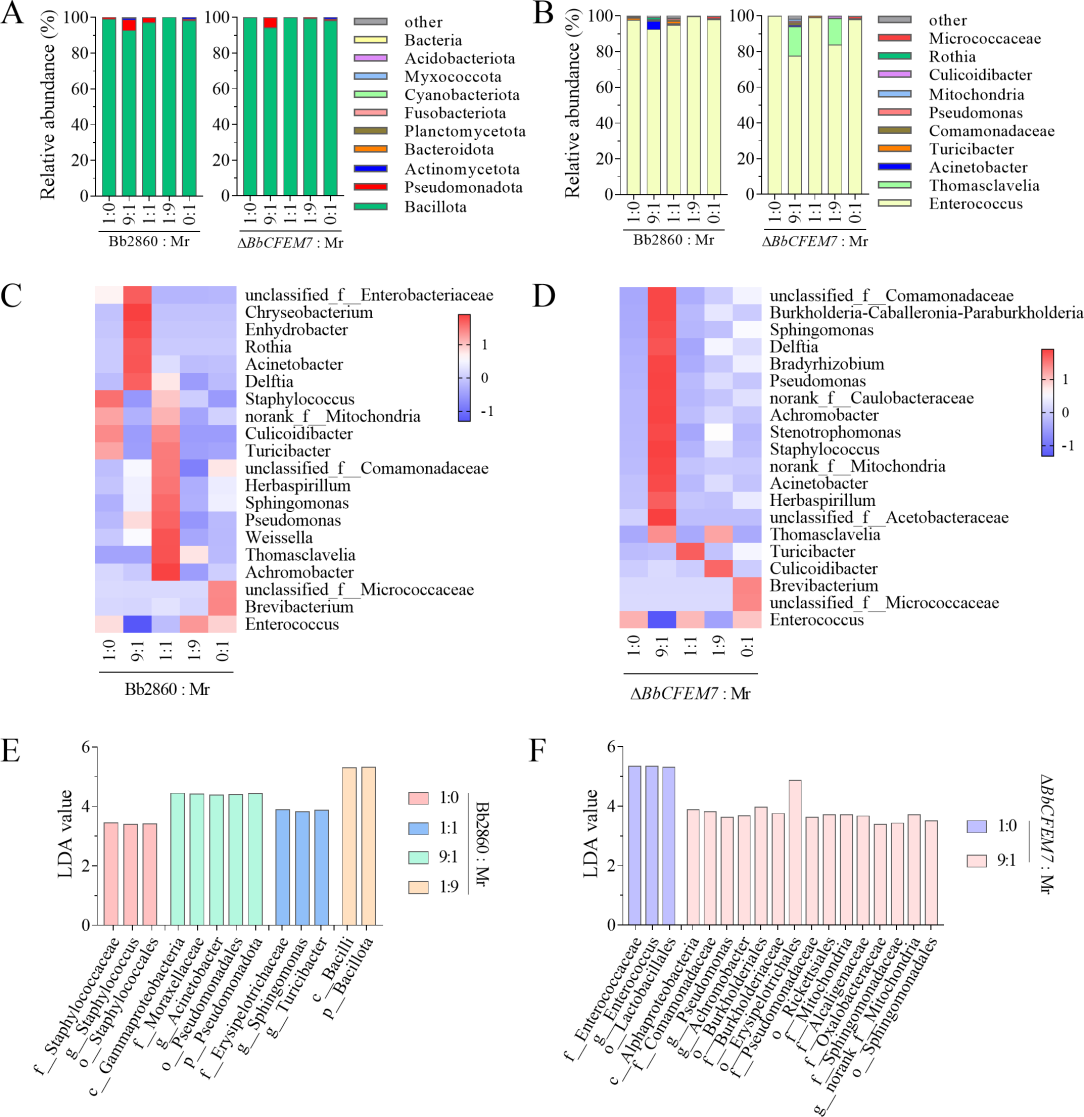
Effects of *BbCFEM7* on gut microbial diversity when *B. bassiana* and *M. rileyi* co-infected *S. litura* larvae. (**A**): Distribution of the top 10 microbial species in the gut of *S. litura* larvae infected by five proportions of 1 × 10^5^ conidia/mL *M. rileyi* and conidial suspensions of *B. bassiana*2860 (WT) or Δ*BbCFEM7* mutant, respectively, at the phylum level. (**B**): Distribution of the top 10 microbial species in the gut of *S. litura* larvae infected with conidial suspensions of *B. bassiana* and the Δ*BbCFEM7* mutant, respectively, at the genus level. (**C and D**): Heat maps of the relative abundance of the top 20 microorganisms in the gut of insects infected by *M. rileyi* and *B. bassiana*2860 (WT) in five different ratios and those infected with *M. rileyi* and the Δ*BbCFEM7* mutant under five different ratios, respectively. (**E and F**): Analysis of the gut microbial abundance spectra of insects infected with different combinations of *M. rileyi*, *B. bassiana*, and the Δ*BbCFEM7* mutant.

The gut microbial community structure at both phylum and genus levels was analyzed in depth during co-infection with *B. bassiana* (both WT and Δ*BbCFEM7* mutants) and *M. rileyi*. The top 10 microbial taxa at different infection rates were identified and compared at the phylum level (Fig. 3A) and genus level (Fig. 3B). At the phylum level, the dominant bacteria in both the WT and *M. rileyi* co-infection group and the Δ*BbCFEM7* mutant and *M. rileyi* co-infection group were Pseudomonadota and Bacillota, among others, with no significant differences between the two groups. At the genus level, Enterococcus was the dominant bacterium in the WT and *M. rileyi* co-infection group. In the 9:1 and 1:9 (Bb2860:Mr) groups, the abundance of Thomasclavelia was significantly increased. Compared to the WT and *M. rileyi* co-infected groups, significant changes in the composition of various gut bacteria were observed in larvae co-infected with the Δ*BbCFEM7* mutant and *M. rileyi* across five groups. For instance, in the Δ*BbCFEM7* mutant-only infection group (1:0), populations of Turicibacter and Culicoidibacter were significantly reduced. In the 9:1 (Bb:Mr) group, the population of Sphingomonas was significantly increased, whereas that of Brevibacterium was significantly decreased. Heat maps of the top 20 microbial taxa (Fig. 3C and D) further highlight the pattern of abundance differences between the WT and mutant groups. Additionally, the microbial abundance profiles (Fig. 3E and F) revealed distinct patterns of microbial community aggregation during co-infection, with significant variations in the gut characteristic microbes of insect hosts infected by *B. bassiana* and *M. rileyi* at different ratios. For example, in the 9:1 (Bb:Mr) group, the Δ*BbCFEM7* mutant and *M. rileyi* co-infected hosts exhibited as many as 15 insect gut microbes compared to the five characteristic microbes in the WT and *M. rileyi* co-infected hosts. The specific effects of *BbCFEM7* on the host gut microbial community during co-infection were elucidated by analyzing the changes in gut microbial species and abundance under various proportional combinations.

### Changes in gut microbiota of *S. litura* infected by *B. bassiana* and *M. rileyi*

**Fig 4:**
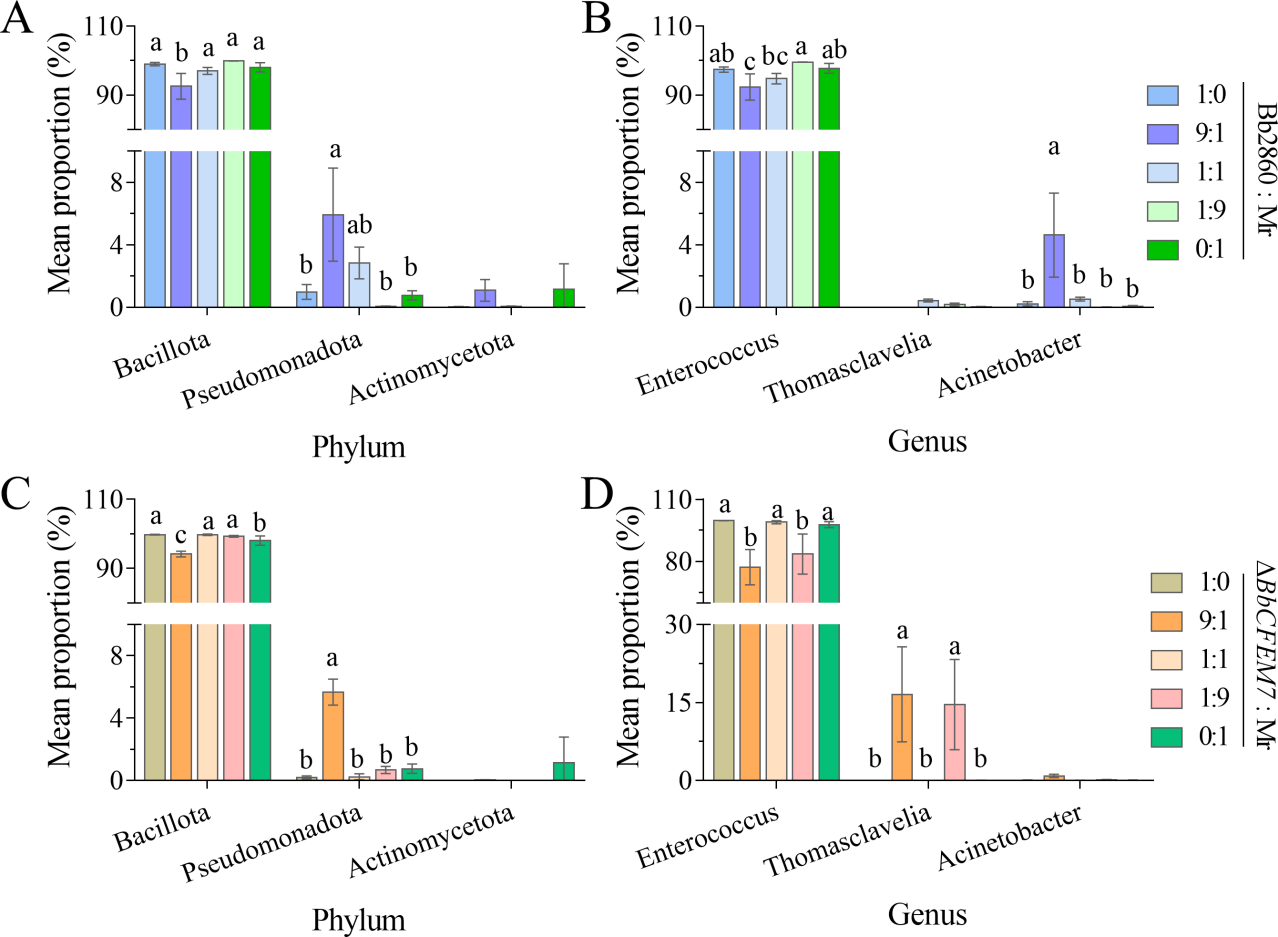
Changes in the gut flora of *S. litura* infected by *B. bassiana* and *M. rileyi*. (**A and B**): Changes in the host gut flora of *M. rileyi* and *B. bassiana*2860 (WT) at the phylum and genus levels, respectively. (**C and D**): The gut flora changes in hosts infected with *M. rileyi* and the Δ*BbCFEM7* mutant at the phylum and genus levels, respectively. Statistical significance was tested using Tukey’s HSD test: *P* < 0.05. The error bars represent the standard deviation.

At the phylum and genus levels, significant changes were observed in the gut microbiome of larvae co-infected with *B. bassiana* (both WT and Δ*BbCFEM7* mutants) and *M. rileyi* (Fig. 4). The results indicated that infection with Bacillus cocciformis by WT strains led to significant changes in the gut microbial community structure compared with the Δ*BbCFEM7* mutant. In the groups with 9:1 and 1:9 ratios, the population of Enterococcus, when co-infected by the Δ*BbCFEM7* mutant and *M. rileyi*, decreased significantly compared with the groups co-infected with the WT and *M. rileyi*. Additionally, the population of Thomasclavelia increased significantly.

### Competition between *B. bassiana* and *M. rileyi in vitro* and *in vivo*

**Fig 5:**
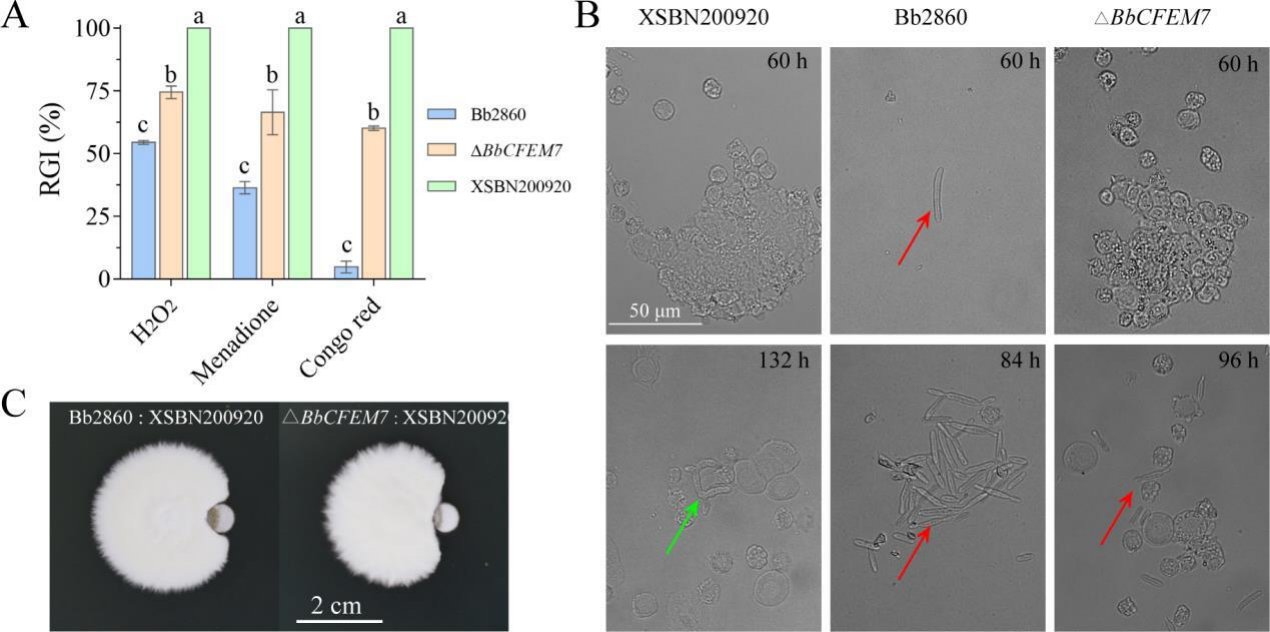
*In vitro* and *in vivo* competition between *B. bassiana* and *M. rileyi*. (**A**): Growth status of *B. bassiana* 2860 (WT), Δ*BbCFEM7* mutant, and *M. rileyi* XSBN200920 on various chemical stress media on day 7. Stressors include Congo red, H_2_O_2_, and menadione. Differences were statistically significant as determined by Tukey’s HSD test: *P* < 0.05. Error bars represent standard deviation. (**B**): Growth of fungal conidia in the hemocoel of *S. litura* larvae was assessed at various time intervals following the injection of a suspension containing 1 × 10^5^ conidia/mL of either *B. bassiana* 2860 (WT), the Δ*BbCFEM7* mutant, or *M. rileyi* XSBN200920. The red arrow indicates *B. bassiana* in the blood cavity, and the green arrow indicates *M. rileyi* in the hemolymph. (**C**): Growth confrontation of M. rileyi with *B. bassiana* 2860 (WT) or Δ*BbCFEM7* mutant on an SDAY plate. The starting point of colony growth is 1.5 cm away. Tukey’s HSD test: *P* < 0.05. Error bars represent standard deviation.

The competitive relationship between *B. bassiana* (WT and Δ*BbCFEM7* mutants) and *M. rileyi* has been confirmed both *in vitro* and *in vivo*. *In vitro* competitive analysis using chemical stress media (Fig. 5A) showed that the Δ*BbCFEM7* mutants were significantly less resistant to stress agents such as Congo red, H_2_O_2_, and menadione compared to the WT strains of *B. bassiana*. In contrast, *M. rileyi* exhibited almost complete loss of resistance to these three stressors at the same concentrations. *In vivo* competitive analysis demonstrated different growth patterns of fungal spores in the hemolymph of caterpillars (Fig. 5B). Compared to the WT, the Δ*BbCFEM7* mutant strain colonized the hemolymph more slowly, with *M. rileyi* displaying the slowest proliferation rate in the host hemolymph. Additionally, antagonistic experiments on SDAY plates (Fig. 5C) showed that *B. bassiana* 2860 (WT) was more effective at inhibiting the growth of *M. rileyi* compared to the Δ*BbCFEM7* mutant, with significant differences observed in colony growth and inhibition zones.

## DISCUSSION

This study analyzed the function of *BbCFEM7* in *B. bassiana* and its influence on host pathogenicity during co-infection with *M. rileyi* in *S. litura*. The results demonstrated that the presence of *BbCFEM7* significantly enhanced the virulence of *B. bassiana* (Fig. 1). Specifically, the WT exhibited a shorter LT_50_ compared to the Δ*BbCFEM7* mutant across all tested ratios. To further validate the role of *BbCFEM7* in natural infection pathways, we performed virulence assays using cuticle immersion treatments (Fig. S1). The LT_50_ values were 7.13 days for the WT strain and 6.97 days for the complementation mutant, whereas the Δ*BbCFEM7* mutant did not achieve 50% cumulative mortality, making LT_50_ calculation unfeasible. This finding directly demonstrates that *BbCFEM7* is crucial in natural infection pathways. It corroborates previous studies that suggest that *BbCFEM7* plays a key role in iron acquisition and the fungal life cycle (12). Given the involvement of CFEM domain proteins in iron ion uptake and utilization and the importance of iron metabolism in fungal growth and pathogenicity, it is plausible that *BbCFEM7* enhances the pathogenicity of *B. bassiana* by regulating iron metabolic pathways. Notably, spore germination assays showed that germination rates for the WT (65.89%) and the complementation mutant (65.65%) were significantly higher than those of the Δ*BbCFEM7* mutant (43.60%) (Fig. S1). This suggests that *BbCFEM7* may also affect conidial germination capacity, thereby providing new insights into its pathogenic mechanism (12).

The gut microbiota of insects plays a crucial role in their host defense system, combating pathogen invasion through multiple mechanisms (18). These include enhancing immune responses, participating in pathogen clearance, metabolic detoxification, and maintaining intestinal homeostasis. Research has shown that in the hindgut of termites, Cononympha leidyi and other symbiotic protozoa participate in the nitrogen cycle and inhibit infections by entomopathogenic fungi through chitin degradation (19). In *Bactrocera dorsalis*, gut microorganisms enhance the expression of AMPs by regulating the host’s Imd signaling pathway, thereby resisting the invasion of foreign pathogens (20). Some gut bacteria secrete AMPs or organic acids that inhibit the reproduction of pathogenic bacteria (21). Additionally, certain gut bacteria can regulate the host’s immune signaling pathways by secreting metabolites, thus enhancing the host’s immune response (22). These mechanisms not only assist insects in coping with pathogens and toxins in their environment but also have significant implications for their health and survival. In response, insect pathogenic fungi have evolved complex genomic strategies to counteract these host defenses, including targeting the humoral immunity of insects (23) and producing antibacterial substances (24) to enhance their survival and pathogenicity within the host. In *M. robertsii*, COA1 conceals cell wall antigenic structures (25), and Fkp1 targets the host enzyme cathepsin CtsK1 (26), facilitating immune evasion. In *B. bassiana*, oosporein enhances fungal survival by suppressing the production of AMPs and the activity of immune cells (27, 28).

This study further examined the influence of *BbCFEM7* on the gut microbiota of *S. litura* during co-infection, as assessed by microbial diversity sequencing (Fig. 3 and 4). In the analysis of gut microbiomes, PCA two-dimensional plots capture only a portion of the total variation in the data. In contrast, LDA models, by integrating information across all dimensions, reveal differences between treatments more sensitively. As a result, the findings from these two analytical approaches are not entirely consistent. The presence of *BbCFEM7* significantly altered the composition and abundance of gut microbes. Notably, infection with the WT strain significantly increased the relative abundance of Enterococcus in the insects, a phenomenon absent in the Δ*BbCFEM7* mutant infection group. This suggests that *BbCFEM7* can reshape the gut microbiome, an effect potentially closely linked to fungal virulence. This linkage has been demonstrated in locust studies where a deficiency in CFEM domain proteins leads to opportunistic pathogen overgrowth and accelerated host mortality (14). Therefore, the gut microbiota disruption induced by the Δ*BbCFEM7* mutant may underlie its reduced virulence, supporting the dual role of *BbCFEM7* in regulating microbial dynamics and pathogenicity. Additionally, other CFEM proteins influence host behavior and immunity; for example, in *M. robertsii*, Mcdc9 triggers antifungal behavioral defenses in flies (13). The reduced virulence of Δ*BbCFEM7* may thus also reflect enhanced host immune responses. Future studies should aim to clarify the molecular mechanisms by which *BbCFEM7* mediates fungus-host interactions.

This study also explores the competitive relationship between *B. bassiana* and *M. rileyi*, particularly in the presence or absence of *BbCFEM7* (Fig. 5). *In vitro* experiments demonstrated that the WT *B. bassiana* exhibited greater tolerance under chemical stress conditions, while the Δ*BbCFEM7* mutant displayed a significant disadvantage. *In vivo* experiments confirmed that the WT strain colonized insect hemolymph more rapidly than the mutant. These findings suggest that *BbCFEM7* may confer competitive advantages to *B. bassiana* against pathogens like *M. rileyi* by enhancing the fungus’s stress tolerance.

Competitive relationships between fungi are common in nature, particularly within insect hosts. These interactions not only directly influence fungal growth and reproduction but may also affect the host’s health status and immune response. For example, certain gut microbes can inhibit the growth of pathogenic bacteria by secreting antimicrobial substances or by activating the host’s immune system (1). In this study, the presence of *BbCFEM7* significantly enhanced the competitive ability of *B. bassiana*, likely due to its role in iron acquisition and stress resistance. Future research should aim to elucidate the specific molecular mechanisms through which *BbCFEM7* participates in fungal competition and systematically assess the potential impact of this competition on host physiology and health. Additionally, although the effects of *BbCFEM7* on fungal competition have been observed *in vitro* and *in vivo*, further studies are needed to explore how this competition manifests in the natural environment.

### Conclusion

In conclusion, this study reveals the importance of *BbCFEM7* in host-pathogen interactions by comprehensively analyzing its role in the pathogenicity of entomopathogenic fungi, gut microbial community structure, and fungal competiftion. These findings provide a new theoretical basis for the ecological study of insect health and pathogen infection and provide a new method for biological control of pests.

## SUPPLEMENTAL MATERIAL

Fig. S1 spectrum.02457-25-s0001.docx Comparison of pathogenicity and conidial germination rates in *Spodoptera litura* infected via cuticle immersion by WT, *ΔBbCFEM7* mutant, and Δ*BbCFEM7::BbCFEM7* mutant.

ASM does not own the copyrights to Supplemental Material that may be linked to, or accessed through, an article. The authors have granted ASM a non-exclusive, world-wide license to publish the Supplemental Material files. Please contact the corresponding author directly for reuse.
