# Supplementary material for: BbCFEM7 plays an important role in the pathogenicity and gut microbial community formation in the co-infection of Beauveria bassiana with Metarhizium rileyi
Source: Microbiol Spectr. 2026 Jan 6;14(2):e02457-25. doi: 10.1128/spectrum.02457-25 (PMC12889123; doi:10.1128/spectrum.02457-25)
Supplement: Fig. S1 — Comparison of pathogenicity and conidial germination rates in Spodoptera litura infected via cuticle immersion by WT, ΔBbCFEM7 mutant, and ΔBbCFEM7::BbCFEM7 mutant. [file spectrum.02457-25-s0001.docx]

Supplementary Figures for

***BbCFEM7* plays an important role in the pathogenicity and gut microbial community formation in the co-infection of *Beauveria bassiana* with *Metarhizium rileyi***

Xu Zhang *et al.*

*Corresponding author: 2021053@ynau.edu.cn (Y-J Peng)

**This file includes:**

Supplementary Text

Figs. S1

Supplementary Figure


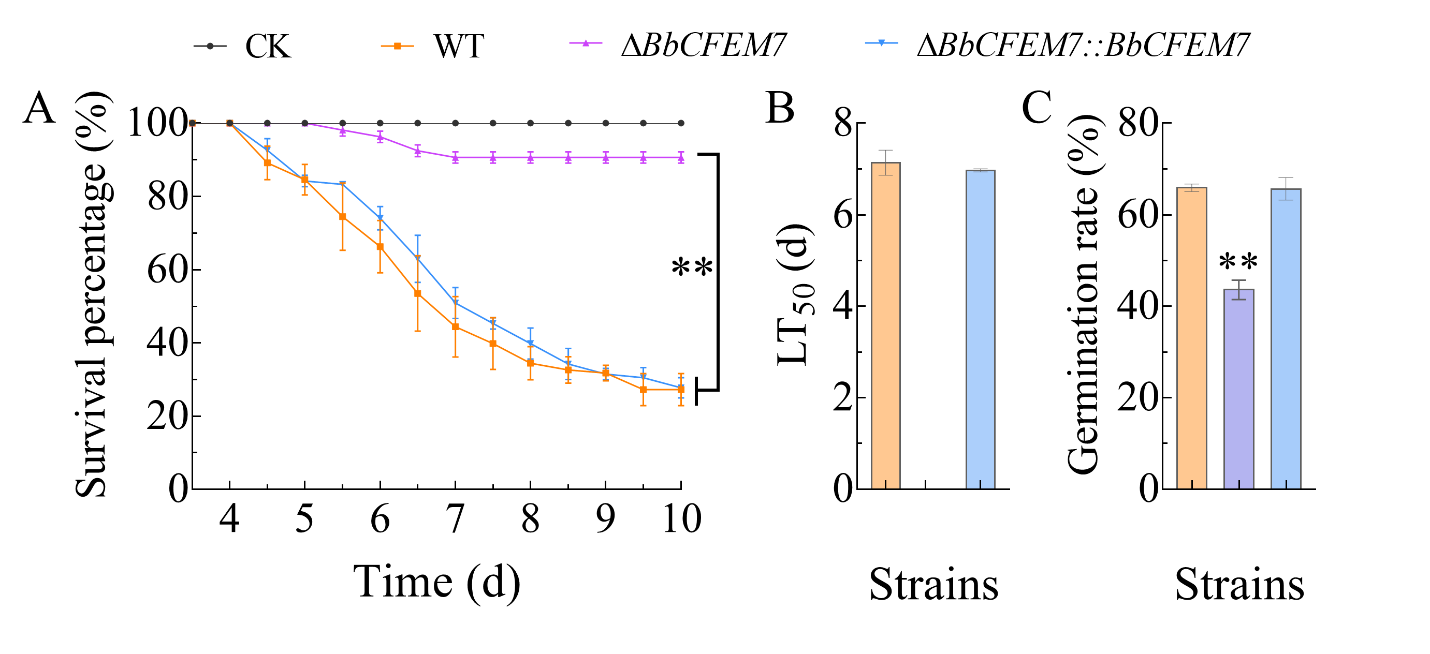


**Figure S1.** Comparison of pathogenicity and conidial germination rates in *Spodoptera litura* infected via cuticle immersion by WT, Δ*BbCFEM7* mutant, and Δ*BbCFEM7::BbCFEM7* mutant. **A** and **B**: Survival curves (**A**) and LT_50_ (**B**) of insects topically infected by WT, Δ*BbCFEM7* mutant, Δ*BbCFEM7::BbCFEM7* mutant, respectively. **C**: Germination rate of conidia from various strains on agar plates in water. Tukey’s honestly significant difference [HSD]: *P* < 0.05. Error bars: standard deviation.
